# Transmission dynamics and control measures of COVID-19 outbreak in China: a modelling study

**DOI:** 10.1038/s41598-021-81985-z

**Published:** 2021-01-29

**Authors:** Xu-Sheng Zhang, Emilia Vynnycky, Andre Charlett, Daniela De Angelis, Zhengji Chen, Wei Liu

**Affiliations:** 1 Centre for Infectious Disease Surveillance and Control, National Infection Service, Public Health England, 61 Colindale Avenue, London, NW9 5EQ UK; 2 Medical Research Council Centre for Outbreak Analysis and Modelling, Department of Infectious Disease Epidemiology, Imperial College Faculty of Medicine, Norfolk Place, London, W2 1PG UK; 3 TB Modelling Group, TB Centre, Centre for Mathematical Modelling of Infectious Diseases and Faculty of Epidemiology and Population Health, London School of Hygiene and Tropical Medicine, London, UK; 4 Medical Research Council Biostatistics Unit, University Forvie Site, Robinson Way, Cambridge, CB2 0SR UK; 5 School of Public Health, Kunming Medical University, Kunming, Yunnan People’s Republic of China

**Keywords:** Applied mathematics, Viral infection, Computational models

## Abstract

COVID-19 is reported to have been brought under control in China. To understand the COVID-19 outbreak in China and provide potential lessons for other parts of the world, in this study we apply a mathematical model with multiple datasets to estimate the transmissibility of the SARS-CoV-2 virus and the severity of the illness associated with the infection, and how both were affected by unprecedented control measures. Our analyses show that before 19th January 2020, 3.5% (95% CI 1.7–8.3%) of  infected people were detected; this percentage increased to 36.6% (95% CI 26.1–55.4%) thereafter. The basic reproduction number (*R*_0_) was 2.33 (95% CI 1.96–3.69) before 8th February 2020; then the effective reproduction number dropped to 0.04(95% CI 0.01–0.10). This estimation also indicates that control measures taken since 23rd January 2020 affected the transmissibility about 2 weeks after they were introduced. The confirmed case fatality rate is estimated at 9.6% (95% CI 8.1–11.4%) before 15 February 2020, and then it reduced to 0.7% (95% CI 0.4–1.0%). This shows that SARS-CoV-2 virus is highly transmissible but may be less severe than SARS-CoV-1 and MERS-CoV. We found that at the early stage, the majority of *R*_0_ comes from undetected infectious people. This implies that successful control in China was achieved through reducing the contact rates among people in the general population and increasing the rate of detection and quarantine of the infectious cases.

## Introduction

An outbreak of severe pneumonia, an infectious disease now named COVID-19, was reported in China in December 2019. The aetiological agent, SARS-CoV-2, a novel coronavirus, was isolated by Chinese authorities on 7th January 2020 and reported by WHO on 9th January 2020. The first case of COVID-19 was reported to have symptom onset on 1st December 2019 in Wuhan city of Hubei province, China^1^, after which the virus quickly spread to other parts of China^2,3^ and the first case outside China was reported on 13th January 2020 (see Table 1). Because of the rapid spread of the disease, WHO announced the outbreak of COVID-19 as a “public health emergency of international concern” on 30th January 2020 and assessed COVID-19 as a pandemic on 11th March 2020^4^. By 25th March 2020, 405,742 cases and 18,791 fatalities associated with COVID-19 had been reported globally, of which, 81,218 cases and 3281 fatalities were reported in mainland China. By 10th April 2020 (25 days later), whilst the corresponding numbers in China had remained relatively unchanged (81,953 cases and 3339 deaths), the global numbers increased dramatically to 1,521,252 cases and 92,798 deaths. The comparatively small increase in the numbers of cases and deaths in mainland China can be attributed to draconian and rapid control measures implemented since late January 2020, starting from the lockdown of the epicentre Wuhan city on 23rd January 2020 and then extending to all of mainland China^3^. These included the complete shutdown and isolation of whole cities, cancellation of Chinese New Year celebrations, and prohibition of school and work attendance, frequent multi-media broadcasts of critical information (e.g., promoting hand washing, mask wearing, and care seeking), massive mobilization of health and public health personnel and military medical units, rapid construction of hospitals for patients with severe symptoms, and reconstruction of shelters for patients with no or mild symptoms. Control measures included travel bans and restrictions, contact reductions and social distancing, screening and contact tracing, early case identification and isolation^2,5^ to curb the epidemic.

**Table 1:** Timeline of outbreak in mainland China.

Date	Event
November 2019	Several pneumoniae of unknown aetiology were discovered in Wuhan city, Hubei province, China
01/12/2019	Symptom onset of the first case reported
31/12/2019	An alert was issued by the Wuhan Municipal Health Commission and a rapid response team was sent to Wuhan by Chinese Centre for Disease Control and Prevention (China CDC) The WHO China Country Office was informed of 27 cases of pneumonia of unknown aetiology detected in Wuhan city, Hubei Province of China
01/01/2020	Wuhan’s Huanan Seafood Wholesale Market, the most probable index source of zoonotic COVID-19 infection, was shut down and disinfected China emergency response team was constructed
02/01/2020	The first 41 confirmed cases were identified as laboratory-confirmed COVID-19 in Wuhan city
03/01/2020	A total of 44 patients with pneumonia of unknown aetiology have been reported to World Health Organisation (WHO) by Chinese authorities Chinese National Health Commission (CNHC) issued the Diagnosis and Treatment plan for pneumonia of unknown aetiology
04/01/2020	One exported case from Wuhan city into Weng Zhou, Zhejiang Province, China
05/01/2020	A total of 59 patients with pneumonia of unknown aetiology have been reported to WHO by the Chinese authorities
07/01/2020	The causative pathogen was identified as a novel coronavirus and genomic characterisation and test method development ensued
12/01/2020	Notification of the novel coronavirus and its sequence was made to WHO
13/01/2020	First exported case was reported in Thailand
17/01/2020	A total of 62 patients with pneumonia of unknown aetiology have been reported
19/01/2020	A total of 198 patients with pneumonia of unknown aetiology have been reported
20/01/2020	Person-to-person transmission was confirmed and announced in China. China’s “National Infectious Disease Law” was amended to make COVID-19 a class B notifiable disease and its “Frontier Health and Quarantine Law” was amended to support the COVID-19 outbreak response effort. By doing this, Chinese law required all cases to be immediately reported to China’s Infectious Disease Information System CNHC started daily situation report of COVID-19 on their official website
23/01/2020	Lockdown of the epicentre Wuhan city, limiting mobility of people in and out of Wuhan, and then the control measures expanded quickly to the neighbouring areas
25/01/2020	The Chinese government raised the response level of the “Preparedness and Response Plan for Novel Infection Disease of Public Health Significance” to the Emergency level, based on the assessment that the risk of health impact caused by COVID-19 on the local population is high and imminent The Chinese government announced its highest-level commitment and mobilized all sectors to respond to the epidemic and prevent further spread of COVID-19
30/01/2020	WHO announced the outbreak of novel coronavirus as a “public health emergency of international concern”
12/02/2020	Definition of confirmed cases for Hubei province was changed to include clinically diagnosed cases or PRC tested positive. The extra high daily number of cases 15,152 was reported from Hubei province On 29/02/2020 this changed back to “clinically diagnosed plus PCR tested positive “
11/03/2020	WHO assessed COVID-19 as a pandemic
17/04/2020	CNHC raised the total death number in Wuhan city from 2579 to 3869

As a novel coronavirus, it is important to understand its transmissibility and severity and to judge how effectively control measures in China have helped stop the spread of SARS-CoV-2 virus which may help inform control of COVID-19 outbreaks in the world and its future potential re-emergence. In previous analyses of transmission dynamics in China, Kucharski et al.^6^ fitted a stochastic transmission dynamic model to four datasets of cases from Wuhan and just considered the period up to 11th February 2020. They estimated that the median daily reproduction number (*R*_t_) in Wuhan declined from 2.35 (95% CI 1.15–4.77) 1 week before travel restrictions were introduced on 23rd January 2020, to 1.05 (95% CI 0.41–2.39) 1 week after. Other modelling studies^2,3,7,8^ also used the confirmed case data from the early pandemic stage to model and estimate the spread, dynamics and control of the COVID-19 outbreak in China. As the number of new confirmed case in mainland China reached a peak only after 17th February 2020 and other relevant data such as those relating to fatality and hospital discharges are becoming available, a further study including more data streams is needed to show the overall effectiveness of the integrated control measures executed in the whole nation.

**Figure 1:**
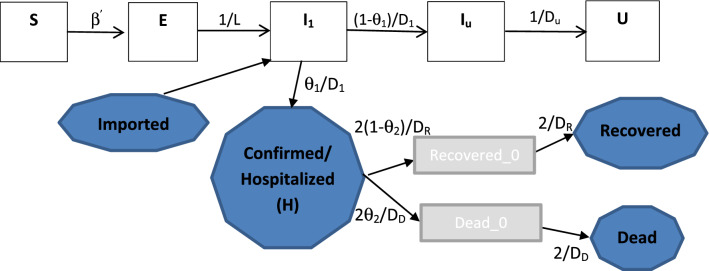
Flow chart of the synthesis model: transmission dynamics and disease reporting processes. The five rectangular boxes represent the hidden transmission dynamics process with *U* representing the number of people who have recovered from an undetected infection and the four blue shaded polygons represent the quantities upon which observations were made. The quantities above the arrows represents the rate at which people go from one compartment to the next and *β*^’^ = $\beta \left( t \right)\left[ {I_{1} \left( t \right)\left( {\theta_{1} +\xi \left( {1 -\theta_{1} } \right)} \right) + \xi I_{u} \left( t \right)} \right]$.

Based on the published data associated with the confirmed cases, death and recovery, in this study we use a Synthesis model (Fig. 1) which covers both the transmission dynamics of the SARS-CoV-2 virus and the disease reporting process of COVID-19 to estimate the transmissibility of SARS-CoV-2 and the severity of COVID-19 in China and the impact on the spread of COVID-19 of control measures the Chinese government has taken from late January 2020.

## Results

The estimates of the model parameters are shown in Table 2. The proportion of COVID-19 cases in mainland China that were detected and reported was about 3.5% (95% confidence interval (CI): 1.7–8.3%) before 19th January 2020, and then increased to 36.6% (95% CI 26.1–55.4%) afterwards. At the early stage before 8th February 2020, the transmissibility of COVID-19 was high with *R*_0_ = 2.33 (95% CI 1.96–3.69); however, it reduced dramatically to 0.04 (95% CI 0.01–0.10) from 8th February 2020 (95% CI 7th–9th February 2020). By fixing the duration from onset of symptoms to death and recovery at 17.8 days and 22.6 days respectively^9^**,** the average infectious period for those that are symptomatic and confirmed and then quarantined is 2.28 days (95% CI 2.01–3.12 days) and the average infectious period for undetected infections is 4.44 days (95% CI 3.91–11.71 days).

**Table 2:** Estimates of model parameters for the COVID-19 outbreak within mainland China.

Name	Definition	Prior	Posterior after distributing 1290 deaths to:	
			Before 20 February	Whole period
*τ*_β_	Time point when the transmission rate is estimated to change because of control measures	U[60,143]*	70.6 (69.2,71.9)	71.4 (70.2,72.0)
*β*_a_	Daily transmission rate before τ_β_	U[0.020,1.00]	0.523 (0.311,0.691)	0.512 (0.292,0.674)
*β*_b_	Daily trasnmission rate after τ_β_	U[0.001,0.20]	0.009 (0.001,0.031)	0.007 (0.001,0.031)
*R*_0,1_	Reproduction number before *τ*_β_	–	2.33 (1.96,3.69)	2.20 (1.85,4.10)
*R*_0_con_	*R*_0_ due to confirmed cases before *τ*_β_	–	0.042 (0.017,0.111)	0.037 (0.013,0.107)
*R*_0_und_	*R*_0_ due to undetected cases before *τ*_β_	–	2.27 (1.92,3.65)	2.16 (182,4.07)
*R*_0,2_	Reproduction number after *τ*_β_	–	0.036 (0.006,0.100)	0.027 (0.006,0.094)
*I*_0_	Initial number of infectious people on 1/12/2019	U[1,500]	8.7 (2.7,49.1)	14.3 (2.4,67.8)
	Initial total number of people infected with the virus on 1/12/2019	–	40.9 (12.8,207.2)	71.1 (12.4,304.4)
*τ*_θ_	Time point when the case ascertainment rate is estimated to change	U[40,62]*	49.3 (47.1,51.6)	50.1 (48.0,52.8)
*θ*_1,a_	Case ascertainment rate before *τ*_θ_	U[1%,25%]	3.48% (1.74%,8.33%)	3.47% (1.35%,8.96%)
*θ*_1,b_	Case ascertainment rate after *τ*_θ_	U[25%,100%]	36.61% (26.07%,55.39%)	38.29% (30.44%,55.67%)
*τ*_F_	Time point when the confirmed case-fatality rate (cCFR) is estimated to change	U[65,95]*	77.0 (75.3,78.0)	77.4 (76.0,78.9)
*θ*_2,a_	Mortality rate among confirmed cases (cCFR_1_) before *τ*_F_	U[0.5%,50%]	9.61% (8.12%,11.36%)	8.56% (7.51%,10.01%)
*θ*_2,b_	Mortality rate among confirmed cases (cCFR_2_) after *τ*_F_	U[0.1%,50%]	0.67% (0.45%,0.97%)	1.28% (0.96%,2.35%)
*D*_1_	Average infectious period before hospitalization (days)	U[2.0,10]	2.28 (2.01,3.12)	2.12 (2.0,2.71)
*D*_1_*+D*_u_	Average infectious period of undetected infections (days)	U[3.0,25.0]	4.44 (3.19,11.71)	4.31 (3.11,13.43)
*η*^HOS^	Dispersion parameter for reported cases	U[1.01,1500]	76.0 (50.2,122.8)	82.8 (53.9,134.1)
*η*^Death^	Dispersion parameter for deaths	U[1.01,5000]	3.5 (2.4,5.2)	4.0 (2.8,5.9)
*η*^Recovery^	Dispersion parameter for recoveries	U[1.01,1500]	144.8 (102.8,205.6)	150.3 (104.7,215.37)
IFR_1_	Infection fatality rate (*θ*_1,a_*θ*_2,a_) before *τ*_θ_	–	0.33% (0.17%,0.85%)	0.30% (0.11%,0.81%)
IFR_2_	Infection fatality rate (*θ*_1,b_*θ*_2,a_) between *τ*_θ_ and *τ*_F_	–	3.51% (2.60%,5.12%)	3.31% (2.47%,4.89%)
IFR_3_	Infection fatality rate (IRF_2_ = *θ*_1,b_*θ*_2,b_) after *τ*_F_	–	0.24% (0.15%,0.41%)	0.50% (0.34%,0.86%)

The 1290 deaths within Hubei province added on 17th April 2020 were distributed over either the period before 17th April (i.e., the whole period) or before 20th February 2020 in proportional to the daily number of deaths reported before 17th April 2020. The relative infectiousness of undetected cases compared to confirmed cases *ξ* = 1 (i.e., both undetected and confirmed infections are of the same infectiousness).

The estimated values of model parameters are nearly the same for both ways of distributing 1290 deaths added on 17th April 2020 by Chinese National Health Commission except for estimates of cCFR_2_ and IFR_3_.

*The epidemic was assumed to start from 1st Dec 2019^1^.

In our model, the basic reproduction number is contributed by two parts: the confirmed cases and undetected infections. Confirmed cases and undetected infections are assumed to be equally infectious in this study; combining with the estimate that only about 3.5% of infections were confirmed and reported at the early stage, this suggests that at the early stage, about 98% of *R*_0_ (Table 2) is due to the undetected infections. If the infectiousness of undetected infections is only half or one third of that of confirmed cases^7^, *R*_0_ in the absence of interventions remains nearly the same, and about 96% of *R*_0_ is due to the undetected infections (Supplementary Table S2.1). This indicates that only isolating confirmed cases and their contacts is not enough to stop the spread and that the main factor that stopped the COVID-19 outbreak in mainland China was the dramatic drop in contact rates among the general population (c.f.^7^).

**Figure 2:**
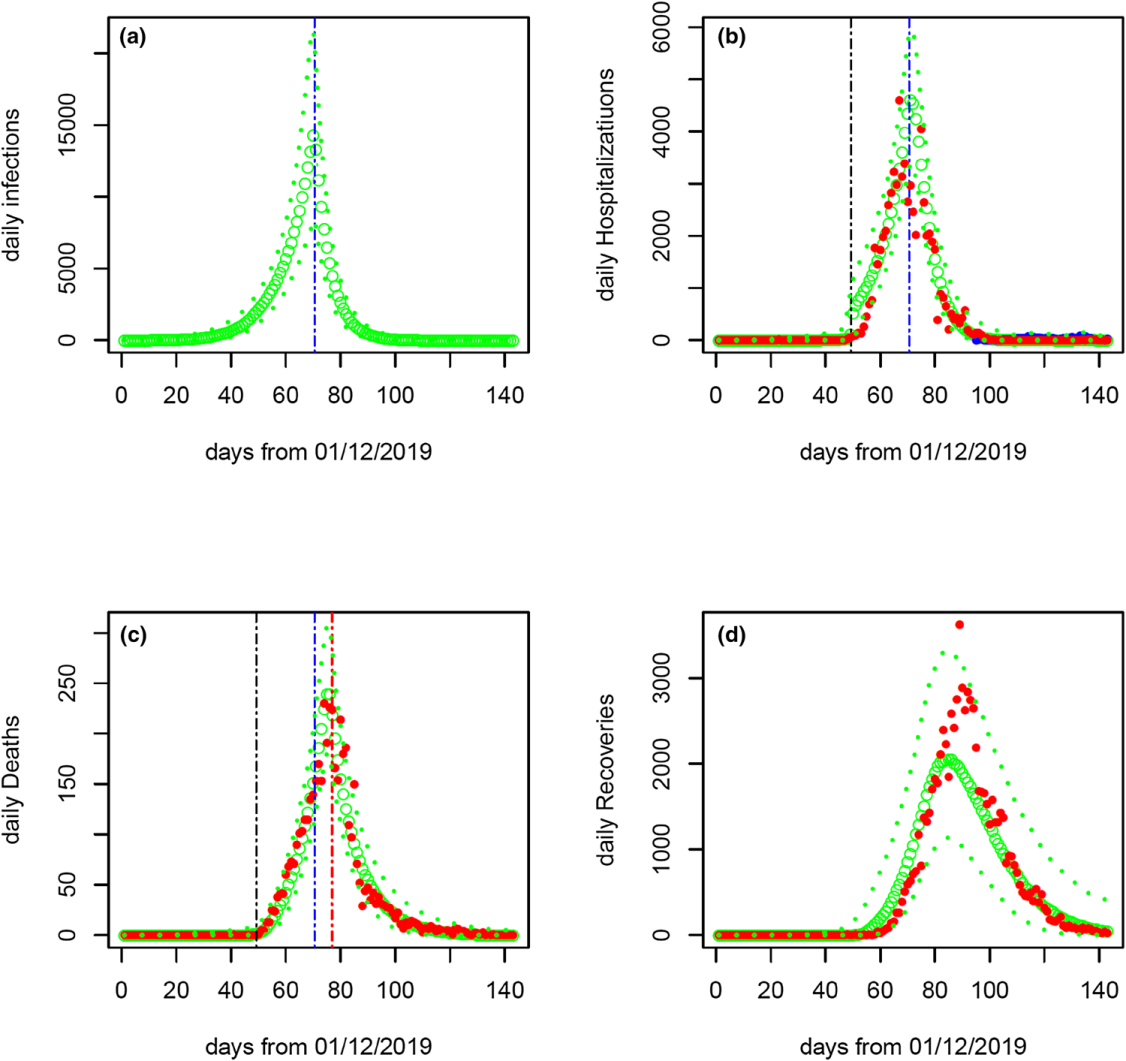
Epidemic curves from 1st December 2019 to 21st April 2020. Model predictions of infections (panel **a**) and model fitting to the daily numbers of confirmed cases (panel **b**), deaths (panel **c**) and recovered cases (panel **d**) are shown. The model predictions are obtained after redistributing the 1290 deaths added to the official data on 17th April 2020 to the period before 20th February 2020 in accordance with the daily number of deaths reported before 17th April 2020. The green dotted lines represent the model predictions (large green circles for median and thin green dotted lines for lower and upper levels of 95% confidence interval). The red points are the observed data. The black, blue and red vertical lines denote the estimates of times when the case ascertainment rate, transmission rate and confirmed case fatality rate respectively are estimated to have changed. In panel (**b**), the blue points represent daily number of imported cases which are nearly invisible. Note that the daily number of confirmed cases (15,152) on 12th February 2020 (day 74) is beyond the range shown in panel (**b**).

The model fitting to the daily number of hospitalizations (confirmed cases), the daily number of deaths, and daily number of recovered people are shown in Fig. 2. Our model analysis suggests that the death rate could have changed around 15 February 2020 (95% CI 14th–16th February 2020). Before this date the confirmed case fatality rate (cCFR) is 9.6% (95% CI 8.1–11.4%), after this date it reduces to 0.7% (95% CI 0.4–1.0%). Based on the estimates of the case ascertainment rate and case fatality rate, the infection fatality rate (IFR) is 0.33% (95% CI 0.17–0.85%) before 19th January 2020 and increases to 3.51%(2.60–5.12%) during the period from 20th January to 15th February 2020; after this, it decreases to 0.24% (95% CI 0.15–0.41%). Our model inference suggests that on 1st December 2019, there were 41 (95% CI 13–207) people infected with SARS-CoV-2 virus. These estimates are based on the assumption that the 1290 deaths added on 17th April 2020 were distributed before 20th February 2020. If we assume that the 1290 deaths were distributed before 17th April 2020, the estimates of most model parameters remain nearly the same except for the death-related quantities (Table 2). For example, after 15 February 2020 cCFR changes from 0.67 to 1.28%, and the IFR from 0.24 to 0.50%.

If the public had been made aware of COVID-19 earlier and the control measures started earlier, the size of outbreak could have been much smaller. This can be quantitatively analysed by assuming the same epidemiological characteristics but moving the start times at which the reporting, transmission and death rates changed. The results are listed in Table 3. It shows that if the control measures started 1 week, 2 weeks, or 3 weeks earlier, about 57% (57%), 81% (82%) and 93% (93%) of the confirmed cases (and deaths) would have been averted. However, if the control measures had started 1 week, 2 weeks, or 3 weeks later, the numbers of the confirmed cases (and deaths) would have increased 2.3-fold (2.3), 5.3-fold (5.4), and 12.4-fold (12.5) across mainland China, respectively.

**Table 3:** Impact of changing the start time of control measures during the outbreak in mainland China on the number of infections, confirmed cases and deaths from 1st December 2019 to 21st April 2020.

Start date	Number of infections	Number of confirmed cases	Number of deaths
2^nd^ Jan 2020	17,536 (4501, 47,147)	5642 (936, 20,617)	301 (33, 1198)
9th Jan 2020	41,856 (15,845, 87,826)	14,156 (4,696, 35,681)	769 (205, 2072)
16th Jan 2020	97,184 (47,351, 171,881)	33,547 (16,736, 64,441)	1,828 (828, 3705)
23^rd^ Jan 2020	223,111 (125,333, 361,357)	78,247 (49,893, 122,761)	4280 (2600, 6964)
30th Jan 2020*	509,036 (301,752, 810,468)	181,200 (126,400, 253,818)	9,936 (6,806, 14,269)
6th Feb 2020*	1,164,089 (668,901, 1,898,473)	420,146 (277,738, 583,748)	23,082 (15,068, 32,260)
13th Feb 2020*	2,671,685 (1,394,762, 4,577,990)	971,916 (564,900, 1,449,202)	53,490 (30,710, 78,960)

*China started its Chinese New Year holidays from 25th January 2020, when the number of contacts made between people became much higher than during the other periods of the year. Hence the estimated numbers of cases are likely to be conservative.

To investigate how the epidemic within the epicentre differs from that over the whole nation, we also obtain estimates of model parameters for Hubei province and Wuhan city (Supplementary Table S3.1). The results show that within the epicentre, the transmissibility and case ascertainment rate differ only slightly. However, there was a large difference in the estimated confirmed case fatality rate. Before the time point *τ*_F_ when the confirmed case-fatality was estimated to have changed (15 February 2020), the estimated cCFR is 9.6% (95% CI 8.1–11.4%) across the whole country, 11.5% (95% CI 10.4–13.5%) over Hubei province, and 15.1% (95% CI 13.3–17.6%) within Wuhan city. However, the estimate of cCFR appears to be nearly the same in all three settings after *τ*_F_. These estimaes imply that the fatality rate before 15 February 2020 is significantly higher at the epicentre than the national average, and many deaths of COVID-19 patients were due to insufficient treatments at the epicentre.

## Discussion

Understanding the transmissibility and severity of the novel coronavirus (SARS-CoV-2) is paramount; understanding how its rapid spread was brought under control in mainland China is of practical importance for other countries now facing ongoing outbreaks of COVID-19. In this study we have studied the transmissibility and control of SARS-CoV-2 in China by using a mathematical model to reconstruct the COVID-19 outbreak in mainland China from 1st December 2019 to 21st April 2020. Our analyses indicate that the SARS-CoV-2 had a basic reproduction number of 2.33 (95% CI 1.96–3.69) in the absence of intervention measures and therefore it is highly transmissible. The fatality rate among those that are symptomatic and confirmed was about 9.6% before 15 February 2020, and then reduced to 0.7%. The draconian control measures taken by the Chinese government from 23rd January 2020 brought the spread of SARS-CoV-2 under control in mainland China. However, it took more than 2 weeks for the effect of control measures to emerge.

Our study shows that the early reporting rate (3.5%) was very low, suggesting that about 96.5% of all infections were undetected prior to 19th January 2020 (c.f.^7,10,11^). This might be mainly due to the limited knowledge and unclear definition of the novel disease^12^. The reporting rate of 36.6% at the late stage reflects increased awareness of the virus and consequently escalating rate of medical help seeking behaviour for respiratory symptoms; however, this rate appears relatively low and suggests that many infections were associated with mild symptoms or were asymptomatic. Analyses by China CDC^13^ using data up to 11th February 2020 estimated that COVID-19 has been mild for 81% of infected people. The high proportion of mild symptomatic or asymptomatic infections was further confirmed by a well-investigated outbreak on the Diamond Princess cruise ship during February 2020: among 696 confirmed cases, 410 (58.9%; c.f.^14^) were asymptomatic^15^.

The actions taken by the Chinese government appear to have stopped the spread of COVID-19 in mainland China. This was achieved under very strict control measures, which dropped the transmissibility, as reflected by the effective reproduction number to 0.04, which is 1.8% of its initial value of *R*_0_ = 2.33 (c.f.^3^). This estimate of *R*_0_ at the early stage is consistent with most previous estimates^3,6–8,10,11,16–22^. We found that the infectious period before isolation for confirmed cases is about 2.3 days, which is comparable with the estimates^23–26^; though it appears to be shorter than that obtained by Tian et al*.*^3^: 5.19 days (95% CI 4.51–5.86 days). Our estimate of the infectious period for infections that were not detected and not quarantined (4.4 days ranging from 3.2 to 11.7 days) agrees with the range identified in previous studies^27,28^ of 1–14 days.

Although increasing the rate of detection and quarantine of symptomatic cases can help reduce the number of sources of infection (c.f.^2^), the main force of transmission is from the undetected cases which contributed to most of the transmissibility of SARS-CoV-2 during the early stage of the outbreak in mainland China (c.f.^7^). Hence it is likely that limiting the mobility of the general population made the biggest contribution to stopping the spread of SARS-CoV-2 virus in mainland China. This may further explain why the draconian control measures implemented in China since the 23rd January 2020 took about 16 days (i.e., delay from 23rd January to 8th February 2020) to influence transmission within community.

The impact of actions taken by the Chinese government is also reflected on the change of mortality associated with COVID-19. Before 15th February 2020, cCFR is estimated at 9.6% and then it reduced to about its 7% (i.e., 0.67%). This huge reduction in cCFR may be due to the significant improvement of medical treatment and availability of medical resources in China, especially in the epicentre Hubei province.

It is interesting to compare our estimate of the mean case fatality rate against that from other studies, which, after averaging the values estimated before and after the 15th February, is 5.14%. This is slightly less than that of Deng et al*.*^29^: 5.65% (95% CI 5.50–5.81%). Including death data up to 17th April 2020, Deng et al*.*^29^ used individual-level data for cases to obtain their estimate which is close to both the corresponding crude or naïve confirmed case fatality risk: 4632/82,758 = 5.60% and the approximator of deaths/(deaths + recoveries) = 4632/ (4632 + 78,112) = 5.60% as of 21st April 2020. Based on the data up to 11th February 2020, Verity et al*.*^9^ suggested that the overall CFR for the outbreak in China was 1.38% (95% CI 1.23–1.53%). Using data up to 29th February 2020, Wu et al*.*^30^ found the overall symptomatic case fatality rate (the probability of dying after developing symptoms) to be 1.4% (95% CI 0.9–2.1%). Russell et al*.*^31^  used several simplifying assumptions to obtain  an estimate of the CFR in China to be 1.2% (95% CI 0.3–2.7). Our estimate of CFR is higher than these, which largely results from the fact that these three studies did not include the 1290 deaths added to the official data on 17th April 2020 by the Chinese National Health Commission^32^.

Through model fitting to observed data in mainland China, we found that the model predicted that there had been twenty eight times more cases than were reported at the early stage of the outbreak; even at the late stage since 19th January 2020, the estimated number of infected people were more than twice those reported. Based on the epidemiological characteristics obtained from COVID-19 outbreak data, our analyses suggest that even if the control measures had started 1 week earlier, they would have averted 57% of confirmed cases and 57% of deaths. If they had started 3 weeks earlier, then 93% confirmed cases and 93% of deaths would have been averted. This estimate of the effect of start time of control measures is smaller than but comparable with what Lai et al*.*^2^ and Yang et al*.*^5^ found, although both studies estimated only the number of infections. These findings highlight the importance of an early response in controlling transmission in the population.

As in Kucharski et al*.*^6^, we assumed the latent period is equal to the incubation period. In view of evidence that there is pre-symptomatic transmission^28,33,34^, it is interesting to know whether this will alter the results of our model. For this, we modified the model system (Fig. 1) by dividing exposure stage *E* into two equal sub-classes *E*_1_ and *E*_2_ and assumed that people in *E*_2_ can transmit the virus with the same infectiousness as ill cases (SI Sect. 4). We obtain similar results (Supplementary Table S4.1): For example, the basic reproduction number before the control measures is 2.22 (95% CI 1.95–2.92) of which 98% was due to undetected cases, and the cCFR is 9.5% (95% CI 8.0–11.2%) before 15 February 2020 and then reduces to 0.7% (95% CI 0.4%, 1.1%). This indicates our conclusions are relatively unaffected by assumptions relating to pre-symptomatic transmission.

The non-pharmaceutical interventions introduced in mainland China appeared to have stopped the spread of the virus, but the risk of another potential outbreak remains. As estimated in our analyses, at most 223,111 individuals were infected up to 21st April 2020 during the outbreak (Table 3), which is less than 0.02% of the Chinese population. Even if all those recovered from infection have developed complete immunity^35^, this level of immunity in the Chinese population is considerably lower than the herd immunity threshold of 55% which is required for control of transmission^36,37^. This simply implies that once the strict quarantine measures currently in place in mainland China are relaxed, transmission of SARS-CoV-2 in China is very likely to rebound, especially given the extent of ongoing transmission across the rest of the world (c.f.^38^). Nevertheless, the non-pharmaceutical interventions appear to have halted the spread of SARS-CoV-2 virus in mainland China and bought time for vaccines and drugs to be developed and used later on.

It is of interest to compare the novel coronavirus SARS-CoV-2 with other two coronaviruses: SARS-CoV-1 and MERS-CoV which caused large outbreaks in human populations. SARS-CoV-1 has a *R*_0_ from 2 to 5^39^ and case-fatality rate of 9.6% among probable cases in mainland China^40^. MERS-CoV in the 2015 outbreak in South Korea has been estimated to have a *R*_0_ from 2 to 7^41^ and case fatality rate of 34.5% among laboratory-confirmed cases^42^. This suggests that that SARS-CoV-2 is nearly as transmissible as SARS-CoV-1 and MERS-CoV, but it is potentially less severe particularly when sufficient treatments are available. Furthermore, there was no evidence of a super-spreader event occurring in any of the Chinese health facilities serving COVID-19 patients, which is distinct from the 2003 outbreak of SARS-CoV and 2015 MERS-CoV outbreak in South Korea. Due to advanced modern transportation, people can move easily and quickly across the world, which allows SARS-CoV-2 to quickly spread to other countries. This is in a sharp contrast with both SARS-CoV-1 and MERS-CoV which have been controlled and confined within relatively limited areas of the world. This sharp difference may be attributed to another aspect of the coronaviruses: a large proportion of SARS-CoV-2 infections are associated with mild symptoms or are asymptomatic^13–15^, while both SARS-CoV-1 and MERS_CoV are highly symptomatic^21,33^. In the absence of high coverage with highly effective vaccines, this characteristic of the SARS-CoV-2 virus will make it challenging for humans to control and manage it.

Compared with previous studies^2,3,5,6,21,22^, our analyses have two key strengths: our investigations are based on three datasets (confirmed cases, deaths and recovered cases) and we model the outbreak over a long period (143 days) which should avoid any bias and confounding arising due to observations over a short period. This study also has several limitations. To model the transmission dynamics and disease reporting, our synthesis model has included several simplifications. To reflect the temporal change in the ascertainment rate, transmissibility and case fatality of COVID-19, two different values are assumed for each. These quantities are likely to change gradually during the outbreak, as is the case for public awareness and interventions^3^. For example, Tsang et al*.*^12^ found the ascertainment rate changed as the case definition for COVID-19 changed from being initially narrow to becoming gradually wider during the period from 15th January to 3rd March. The time-to-event intervals such as the delay from symptom onset to death may also change as epidemic grows^9,24,26^ and the time from symptom onset to hospitalization may reduce as more reliable medical resources become available^22,24^. Further, in this study we ignore the heterogeneity in both geography and age^3,7,13^. To provide more specific and practically useful information for control measures, it needs to look at variations between regions^3,7^ and in different age groups^13^. A further limitation is that we model the overall effectiveness of integrated intervention measures rather than the different types of control measures and therefore cannot provide insight into their relative impacts in stopping the spread of infection (c.f.^2,3,16^).

In conclusion, our finding that the main driver of transmission of SARS-CoV-2 at the early stage of the outbreak in mainland China came from the undetected infections provides helpful information for policy makers when designing optimal intervention strategies. In the absence of vaccination and effective drugs, early detection and isolation are essential for containing and controlling the spread of SARS-CoV-2 but reducing contact rates among people in the general population is likely to have the biggest impact.

## Methods

### Data

We extracted the following data relating to COVID-19 for mainland China from 3 datasets on the website of Chinese National Health Commission for the period from 1st December 2019 to 21st April 2020: the daily number of confirmed cases who were confirmed/admitted to hospital, the daily number of deaths and the number of patients who had recovered each day. Here the data are given by the symptom onset date during the period 1st December 2019–1st January 2020 from^1^ and by reporting date thereafter from the website of Chinese National Health Commission, due to difficulties in collecting the onset dates of those cases through the website. The reporting date is assumed to be the same as the date that cases were diagnosed. Before 12th February 2020, confirmed cases were defined as those who were positive for SARS-CoV-2 on PCR; from 12th February 2020 confirmed cases were defined (for the epicentre Hubei province) as those who were either clinically diagnosed or positive for SARS-CoV-2 on PCR^12^, and as those who were PCR positive for SARS-CoV-2 for the rest of China. This case definition resulted in the number of confirmed cases increasing from 2015 to 15,151 between the 11th and 12 February 2020.

The daily numbers of reported deaths were adjusted to account for changes to the numbers of deaths that were made by the Chinese government, as follows. Specifically, on 17th April 2020, Chinese National Health Commission revised its estimates of the total numbers of COVID-19-related deaths in Wuhan city that had occurred since the start of the outbreak from 2579 to 3869^32^. It stated that these additional 1290 deaths probably occurred before 20th February 2020, given that, after this date, the number of hospitals that could treat COVID-19 patients increased from 2 to 48, and the HOUSHEN and LEISHEN hospitals, and FANGCANG shelters that have been constructed since then also provided an increased number of beds meeting the needs of COVID-19 patients with different symptoms. Furthermore, the system of data collection improved rapidly, and the number of missed cases/deaths decreased greatly during the course of the outbreak. As detailed information for these 1290 deaths is not available, for the sake of model fitting, these 1290 deaths were distributed in proportion to the number of deaths reported each day from the date of the first death (10th January 2020) to 20th February 2020. For example, the number of deaths reported on 18th January 2020 was 26, the number is now corrected to 26 + 26 × 1290/2236 = 26 + 15 = 41. Here 2236 is the cumulative number of deaths reported up to 20th February 2020 before the government had revised its estimates in April. In sensitivity analysis, we also explored the effect of assuming that these 1290 deaths added on 17th April 2020 were distributed in proportion to the daily number of deaths reported over the entire period from 10th January to 17th April 2020. Except for estimates of mortality rate,  the results shown in Table 2 (and described in the “Results” section) are very similar for both ways of distributing the 1290 deaths added to the official statistics on 17th April 2020.

### Model

In this study we use a synthesis model^43^ (Fig. 1), which combines the hidden transmission dynamics of SARS-CoV-2 virus and the reporting system of COVID-19, to investigate the transmissibility and severity of COVID-19 and the efforts to contain and control the spread of SARS-CoV-2. We assume the transmission dynamics of SARS-CoV-2 virus are described by an SEIR compartmental model, with a few modifications, as shown in Fig. 1. That is, a susceptible person (*S*) can contract SARS-CoV-2 virus from infectious people and then enter the latent class (*E*); after an average latent period (*L*), the exposed person progresses to become infectious (*I*_1_). A fraction (*θ*_1_) of these people (with severe symptoms) will be detected and admitted to hospital (*H*) and will then be treated and isolated from the community, with a fraction (1 − *θ*_2_) recovering after an average period *D*_R_ and the other fraction dying after an average period *D*_D_. The other fraction (1 − *θ*_1_) of infected people, typically with mild or asymptomatic infections (*I*_u_) will not be detected and hence remain in the community as sources of infection for a further average period *D*_u_ before recovering. For simplicity, we assume that the average latent period equals the incubation period, which is fixed at *L* = 5.2 days as estimated by^18,26,44^. SARS-CoV-2 virus can be transmitted by three possible modes: respiratory transmission (through respiratory droplets when symptomatic people sneeze or cough), aerosol transmission (through fine virus particles that were aerosolized) and contact transmission (through contacting the contaminated surface). For simplicity and consistency with these easy and quick modes of transmission, we assume that people mix randomly. Although China is a large country with a population of 1,400,050,000 people residing on a huge area of 9,596,960 km^2^, its recent urbanization and development of rapid transport systems make it easy and quick for people to move around the country. This makes it reasonable to model the transmission of SARS-CoV-2 within the whole country as a well mixing population. For comparison, we also model the spread of COVID-19 within Hubei province and Wuhan city where the outbreak started (SI Sect. 3).

The synthesis model is described by Eq. (1). The 9 compartments are defined in Table 4 and the definitions of model parameters are given in Table 2.

1 $$\begin{aligned} & \frac{d}{dt}S\left( t \right) = - \beta \left( t \right)S\left( t \right)\left( {I_{1} \left( t \right)\left( { \theta_{1} + \xi \left( {1 - \theta_{1} } \right)} \right) + \xi I_{u} \left( t \right)} \right)/N \\ & \frac{d}{dt}E\left( t \right) = \beta \left( t \right)S\left( t \right)\left( {I_{1} \left( t \right)\left( { \theta_{1} + \xi \left( {1 - \theta_{1} } \right)} \right) + \xi I_{u} \left( t \right)} \right)/N - E\left( t \right)/L \\ & \frac{d}{dt}I_{1} \left( t \right) = E\left( t \right)/L - I_{1} \left( t \right)/D_{1} + Imported\left( t \right) \\ & \frac{d}{dt}I_{u} \left( t \right) = \left( {1 - \theta_{1} } \right)I_{1} \left( t \right)/D_{1} - I_{u} \left( t \right)/D_{u} \\ & \frac{d}{dt}H\left( t \right) = \theta_{1} I_{1} \left( t \right)/D_{1} - 2\theta_{2} H\left( t \right)/D_{{{\text{D}}}} - 2(1 - \theta_{2} )H\left( t \right)/D_{{{\text{R}}}} \\ & \frac{d}{dt}Recovered\_0\left( t \right) = 2(1 - \theta_{2} )H\left( t \right)/D_{{{\text{R}}}} - 2Recovered\_0\left( t \right)/D_{{{\text{R}}}} \\ & \frac{d}{dt}Recovered\left( t \right) = 2Recovered\_0\left( t \right)/D_{{{\text{R}}}}  \\ & \frac{d}{dt}Dead\_0\left( t \right) = 2\theta_{2} H\left( t \right)/D_{{{\text{D}}}} - 2Dead\_0/D_{{{\text{D}}}} \\ & \frac{d}{dt}Dead\left( t \right) = 2Dead\_0\left( t \right)/D_{{{\text{D}}}} \\ \end{aligned}  $$

**Table 4:** Definitions of model compartments.

Name	Definition
**Transmission dynamics**	
*S*(*t*)	Number of susceptible people at time *t*
*E*(*t*)	Number of exposed (infected but not yet infectious) people at time *t*
*I*_1_(*t*)	Number of all infectious people at time *t* who have not yet been detected
*I*_u_(*t*)	Number of infectious people at time *t* who have not been detected and will remain undetected
**Disease reporting**	
*H*(*t*)	Number of people who were hospitalized/reported due to COVID-19 at time *t*
*Dead*_0(*t*)	Number of people in the “Dead_0” compartment. This is defined as an intermediate compartment between those hospitalised (*H*) and *Dead* at time *t*. This compartment does not have any epidemiological interpretation, but including it in the model results in the distribution of the time interval from hospitalisation to death following the Gamma distribution
*Dead*(*t*)	Number of people who have died from COVID-19 at time *t*
*Recovered*_0(*t*)	Number of people in the “Recovered_0” compartment. This is defined as an intermediate compartment between those hospitalised (*H*) and *Recovered* at time *t.* This compartment does not have any epidemiological interpretation, but including it in the model results in the distribution of the time interval from hospitalisation to recovery following the Gamma distribution
*Recovered*(*t*)	Number of recovered people at time *t*

Here *N* = 1,400,050,000 is the total population size in mainland China and is assumed to be constant during the outbreak. The model also includes imported cases (i.e., *Imported*(*t*) in equation for *I*_1_) from outside China as reported by Chinese National Health commission since 24th March 2020, which is likely to help with improving the reliability of our estimates of the transmissibility at the late stage of the epidemic and the effectiveness of intervention measures. Including the numbers of imported cases in the model means that we implicitly include people entering China at the latent stage. For example, due to the stronger and improved testing and tracing services at the late stage of the epidemic, they would have been put into isolation for 2 weeks after entering China and would have been detected as cases during that time.

Consistent with our assumption that people mix randomly, the force of infection (the rate at which susceptibles are infected per unit time) is assumed to be proportional to the number of infectious people at time *t*, which is given by the following expression in the equation for the rate of change in the number of susceptible individuals (i.e. d*S*(*t*)/d*t*):

$$\left( {I_{1} \left( t \right)\left( {\theta_{1} + \xi \left( {1 -\theta_{1} } \right)} \right) + \xi I_{u} \left( t \right)} \right)$$

This expression comprises three terms: *I*_1_(*t*)*θ*_1_—the number of infectious people at time *t* who have severe symptoms and will be detected and hospitalised, *I*_1_(*t*)(1 − *θ*_1_)—the number of infectious people who have mild or no symptoms and will not be detected and therefore will transfer to the compartment *I*_u_, and *I*_u_(*t*)—the number of infectious people who have not been detected and will remain so. The parameter *ξ* is the relative infectiousness of people with undetected infections compared to confirmed cases.

To model the potential changes in the transmission rate (the rate at which two specific people come into effective contact per unit time) due to the combined and dramatic control measures taken by Chinese government, we simply assume that the transmission coefficient, *β*, differed before and after they are introduced as shown in the Eq. (2)

2 $$\beta \left( t \right) = \left\{ {\begin{array}{*{20}c} {\begin{array}{*{20}c} {\beta_{a} } & {t \le {\tau_{\beta} } } \\ \end{array} } \\ {\begin{array}{*{20}c} {\beta_{b} } & {t >{\tau_{\beta }} } \\ \end{array} } \\ \end{array} } \right.$$

(c.f.^3,41^). Here *τ*_β_ is the time point when the transmission rate changed as a result of the interventions and will be estimated in our analyses.

In addition, the limited knowledge and unclear definition of COVID-19 in the early stages of the outbreak means that the proportion of cases that were detected and reported was probably low and increased as knowledge about COVID-19 improved and the availability of advanced techniques to test SARS-CoV-2 increased^12^. In particular, case-finding, diagnosis and reporting have sped up since 20th January 2020. Local governments across China encouraged and supported routine screening and quarantine of travellers from Hubei Province in order to identify COVID-19 infections as early as possible^2^. To reflect these changes, we assume that the proportion of people that were detected and reported (*θ*_1_) (or rate of case ascertainment) varied with time as follows

3 $$\theta_{1} = \left\{ {\begin{array}{*{20}c} {\begin{array}{*{20}c} {\theta_{1,a} } & {t \le \tau_{\theta } } \\ \end{array} } \\ {\begin{array}{*{20}c} {\theta_{1,b} } & {t > \tau_{\theta } } \\ \end{array} } \\ \end{array} } \right.$$

Here τ_θ_ is the time point when the rate of case ascertainment changed.

It is also likely that the case fatality rate changed over time, as treatment improved with increasing provision of medical resources. For simplicity, we introduce a time point τ_F_ so that before that time, the confirmed case fatality rate is *θ*_2,a_ and after this time point it becomes *θ*_2,b_, as follows:

4 $$\theta_{2} = \left\{ {\begin{array}{*{20}c} {\begin{array}{*{20}c} {\theta_{2,a} } & {t \le_{F} } \\ \end{array} } \\ {\begin{array}{*{20}c} {\theta_{2,b} } & {t > \tau_{F} } \\ \end{array} } \\ \end{array} } \right.$$

*θ*_2,a_ and *θ*_2,b_ and τ_F_ will be estimated in our analyses.

Although there may have been some sporadic cases of COVID-19 that might not have had the chance to be hospitalized and so would die at home, especially at the early stage of the outbreak, our assumed flow of confirmed COVID-19 patients should approximate actual procedures during the outbreak in mainland China reasonably well^45^. In this study we fix the average durations from onset of symptoms to death and to hospital discharge at (*D*_1_ + *D*_D_ =) 17.8 days and (*D*_1_ + *D*_R_ =) 22.6 days, respectively, consistent with the estimates^9^ from the China outbreak data. To allow the durations from hospitalization to death and from hospitalization to recovery to follow the Gamma distribution rather than the usual exponential distribution^46^, we introduce the intermediate compartments *Dead*_0, and *Recovered*_0. As we use (mostly) data of reported dates, the time-event-length (duration) should be thought to include such reporting delay, which is not explicitly treated in this study^6^.

The basic reproduction number *R*_0_, which is defined as the average number of secondary infectious people generated by an infectious person introduced into a completely susceptible population, is an important quantity which characterises the transmissibility of infectious agents^36^. We can obtain the expression for *R*_0_ by considering the situation without imported cases and obtain the steady-state solution of the equation system (1), with its S^*^ (the size of the population susceptible to infection at equilibrium) being given by

$$S^{*} = N\frac{{1/D_{1} }}{{\beta_{{\text{a}}} [\theta_{{1,{\text{a}}}} + \xi \left( {1 - \theta_{{1,{\text{a}}}} } \right) + \xi \left({1-\theta_{1,{\text{a}}}}\right) D_{{\text{u}}} /D_{1} ]}}$$.

At the steady state, *R*_0_ × (S^*^/*N*) = 1^36,37^, the above expression gives the basic reproduction number at the early stage as follows

5a $$R_{{0,{1}}} = \, \left( {\theta_{{{1},{\text{a}}}} D_{{1}} + \xi ({1} - \theta_{{{1},{\text{a}}}} )\left( {D_{{1}} + D_{{\text{u}}} } \right)} \right)\beta_{{\text{a}}}$$

Similarly, at the late stage after the time point defined as max (*τ*_β_, *τ*_θ_), the effective reproduction number under interventions is

5b $$R_{{0,{2}}} = \, \left( {\theta_{{{1},{\text{b}}}} D_{{1}} + \xi ({1} - \theta_{{{1},{\text{b}}}} )\left( {D_{{1}} + D_{{\text{u}}} } \right)} \right)\beta_{{\text{b}}}$$

From Eq. (5a), the contribution from undetected infections to the transmissibility at the early stage is

5c $$R_{{0,{1}}} \left( {\text{undetected cases}} \right) \, = \xi ({1} - \theta_{{{1},{\text{a}}}} ) \, \left( {D_{{1}} + D_{{\text{u}}} } \right)\beta_{{\text{a}}}$$

Here *D*_1_ + *D*_u_ is the average infectious period of the infected people who were not detected. The parameter *ξ* is introduced to measure the relative infectiousness of undetected infections to confirmed cases, and it will take the value of 1.0 (i.e., both undetected and confirmed infection are of the same infectiousness). The above equations for the basic reproduction number can also be obtained using the Next Generation Matrix approach described in Diekmann et al.^47^ (see SI Sect. 5).

To see how the relative infectiousness affects our findings, estimates of *R*_0_ and other model parameters for *ξ* = 1/2, 1/3 are given in Supplementary Table S2.1.

#### Initial seeding

In this study we model the transmission process from 1st December 2019 with initial infections of *I*_1_(0) = *I*_0_ which is to be estimated from model fitting to data (see below). The initial values of the other variables were obtained from *I*_0_ and the total population size by assuming that the epidemic around 1st December 2019 was at its early exponential growth stage, as described in SI Sect. 1. In contrast, Wu et al*.*^21^ assumed that the epidemic during 1st–31st December 2019 was seeded by a constant zoonotic force of infection that caused 86 cases (twice the number of 43 confirmed cases with zoonotic exposure) per day before market closure on 1st January 2020. Kucharski et al*.*^6^ assumed the outbreak started with a single infectious case or 10 cases on 22nd November 2019.

### Inference method

We denote the set of model parameters to be inferred as Θ = {*I*_0_, *β*_a_, *β*_b_, *τ*_β_, *θ*_1,a_, *θ*_1,b_, *τ*_θ_, *θ*_2,a_, *θ*_2,b_, *τ*_F_, *D*_1_, *D*_u_} which is listed in Table 2. For each set of parameter values, the Runge–Kutta fourth order method was used to solve the model equations and to obtain model predicted time series of infections, confirmed cases, deaths, and recovered cases. In the inference of model parameters, directly observed dataset of confirmed/hospitalized/reported cases (denoting it as HOS for short in the following), Death and Recovery are used as illustrated in the following. To capture the large dispersion in the daily numbers of these observations, the negative binomial likelihood function was assumed. The likelihood for number *x*^C^ (*t*) of observations on day *t* is given as

6a $$l(x^{C} \left( t \right)|{\Theta },\eta^{C} ) = \frac{{{\Gamma }\left( {x^{C} \left( t \right) + r^{C} \left( t \right)} \right)}}{{{\Gamma }\left( {r^{C} \left( t \right)} \right){\Gamma }\left( {x^{C} \left( t \right) + 1} \right)}}\left( {\frac{1}{{\eta^{C} }}} \right)^{{r^{C} \left( t \right)}} \left( {1 - \frac{1}{{\eta^{C} }}} \right)^{{x^{C} \left( t \right)}}$$

where

6a $$r^{C} \left( t \right) = \frac{{\mu^{C} \left( t \right)}}{{\eta^{C} - 1}}$$

Here $\eta^{C}$ is the dispersion parameter, which is estimated and *μ*^C^(*t*) are the predictions of the number of hospitalisations, deaths or newly recovered people, as appropriate on day *t* from synthesis model (1). Superscript *C* represents three different datasets: HOS, Death and Recovery.

Special attention was paid to the extra high daily number of cases (15,152) on 12th February 2020 (day 74 from 1st December 2019) due to the change in the case definition in Hubei province^12,48^. In principle, most of these cases might have been accumulated over the days before 12th February 2020. To deal with this complexity, only the cumulative numbers of cases on the 12th February and daily numbers of cases after that will be used in the model inference. Let the reported daily number of HOS be represented by *x*(1), *x*(2), …, *x*(*T*), with *T* being the number of days from 1st December 2019 to 21st April 2020. The likelihood of the cumulative number of cases on 12th February $X = \sum\nolimits_{t = 1}^{74} x \left( t \right)$ is assumed to be

7 $$L_{74} = \frac{{{\text{exp}}\left( { - M} \right)}}{X!}{\text{\rm M}}^{X}$$

Here ${\text{M}} = \sum\nolimits_{t = 1}^{74} {\mu^{{{\text{HOS}}}} } \left( t \right)$ represents the cumulative number of confirmed cases predicted by model. Assuming that the observed daily number of deaths: *y*(1), *y*(2),…,*y*(*T*) and daily number of recovered people: *z*(1), *z*(2),…,*z*(*T*) are conditionally independent, the total likelihood given model parameters Θ is

8 $$L\left( {{\Theta },\eta^{{{\text{HOS}}}} ,\eta^{{{\text{Death}}}} ,\eta^{{{\text{Recovery}}}} } \right) = L_{74} \times \mathop \prod \limits_{t = 75}^{T} l\left( {x\left( t \right){ {|\Theta }},\eta^{{{\text{HOS}}}} } \right) \times \mathop \prod \limits_{t = 1}^{T} l\left( {y\left( t \right){ {|\Theta }},\eta^{{{\text{Death}}}} } \right)l\left( {z\left( t \right){ {|\Theta }},\eta^{{{\text{Recovery}}}} } \right)$$

*Parameter inference*: We assume the uninformative prior distributions *f*(**Θ**) which are uniform for parameters (Table 2). Employing a Bayesian framework through the combination of the prior distribution *f*(**Θ**) and the likelihood *L*(**Θ**,*η*^HOS^*, η*^Death^*, η*^Recovery^_*;*_* x, y, z*), the posterior distribution can be obtained by Markov Chain Monte Carlo simulations (MCMC)^49^. From these samples, we obtain the medians and the 95% confidence intervals for the model parameters. The posteriors of the model parameters will provide the estimates of the transmissibility and the severity of SARS-CoV-2 in mainland China and the effects on transmissibility of control measures implemented by the Chinese government. The same inference method will also be used when modelling the outbreak in epicentre Hubei province and Wuhan city.

To explore the effect of the timing of control measures, we calculate the numbers of infections, cases and deaths that would have occurred if the control measures had been implemented 1, 2, or 3 weeks earlier than they had been, or if they had been implemented 1, 2, or 3 weeks later than they had been. To do this, we simply change the start times at which the reporting, transmission and death rates changed while the transmission parameters, ascertainment rate and fatality rate remain unchanged.

## Supplementary Information

Supplementary Information
