# Supplementary material for: Transmission dynamics and control measures of COVID-19 outbreak in China: a modelling study
Source: Sci Rep. 2021 Jan 29;11:2652. doi: 10.1038/s41598-021-81985-z (PMC7846591; doi:10.1038/s41598-021-81985-z)
Supplement: Supplementary file 1 — Supplementary Information [file 41598_2021_81985_MOESM1_ESM.docx]

**Transmission dynamics and control measures of COVID-19 outbreak in China: a modelling study**

Xu-Sheng Zhang, Emilia Vynnycky, Andre Charlett, Daniela de Angelis, Zhengji Chen, and Wei Liu

**Supplementary information**

**S1 Seeding of infections during the outbreak of COVID-19 in mainland China**

We simulate the spread of COVID-19 from 1^st^ December 2019 with initial number of infectious cases *I*_1_(0) = *I*_0_. Human-to-human transmission has occurred among close contacts since December 2019 (Huang *et al.* 2020). By assuming that epidemic around 1^st^ December 2019 is at its early exponential growth stage with a growth rate *Ψ*_r_, we have

$$\frac{d}{dt}\left( \begin{matrix} E \\ I_{1} \\ I_{u} \end{matrix} \right)=\left( \begin{matrix} -\frac{1}{L} & \left[ {}_{1}+\left( 1-{}_{1} \right) \right]\beta& \beta\\ \frac{1}{L} & -\frac{1}{D_{1}} & 0 \\ 0 & \frac{\left( 1-{}_{1} \right)}{D_{1}} & -\frac{1}{D_{u}} \end{matrix} \right)\left( \begin{matrix} E \\ I_{1} \\ I_{u} \end{matrix} \right)=\psi_{r}\left( \begin{matrix} E \\ I_{1} \\ I_{u} \end{matrix} \right)$$

The infectious status at 1^st^ December 2019 can be approximated as

$I_{1}\left( t \right){-I}_{1}\left( 0 \right){{}_{0}I}_{1}\left( 0 \right)={(E\left( 0 \right)}/L-\frac{I1\left( 0 \right)}{D_{1}})t$

$I_{u}\left( t \right){-I}_{u}\left( 0 \right){{}_{0}I}_{u}\left( 0 \right)=\left( \frac{\left( 1-{}_{1} \right)I1\left( 0 \right)}{D_{1}}-{I_{u}\left( 0 \right)}/{D_{u}} \right)t$

${}_{0}U\left( 0 \right)={(I_{u}\left( 0 \right)}/{D_{u})t}$

Here α_0_= exp(δt*Ψ*_r_)-1 for a small time step δ*t* and *U*(0) is the total initial number of undetected cases who have recovered. From these equations, we have

$$E\left( 0 \right)=\frac{t/{D_{1}}+{}_{0}}{t/L}I_{1}\left( 0 \right)$$

$I_{u}\left( 0 \right)=\frac{\left( 1-1 \right)t/{D_{1}}}{t/{D_{u}}+{}_{0}}I_{1}\left( 0 \right)$

$$U\left( 0 \right)=\frac{t/{D_{u}}}{{}_{0}}I_{u}\left( 0 \right)$$

and *S*(0) = *N*-*E*(0)-*I*_1_(0)-*I*_u_(0)-*U*(0).

Considering the eigenvalue equation of initial exponential growth of infections of transmission dynamics (1) in the main text (Wearing *et al.* 2005),

$\left| \begin{matrix} -\frac{1}{L}-\psi_{r} & \left[ {}_{1}+\left( 1-{}_{1} \right) \right]\beta& \beta\\ \frac{1}{L} & -\frac{1}{D_{1}}-\psi_{r} & 0 \\ 0 & \frac{\left( 1-{}_{1} \right)}{D_{1}} & -\frac{1}{D_{u}}-\psi_{r} \end{matrix} \right|$= 0

we find that the initial growth rate *Ψ*_r_ is linked to the transmission rate *β*_a_ through the following expression:

$\frac{\beta_{a}}{L}\left( \frac{1-{}_{1}}{D_{1}}+\left[ {}_{1}+\left( 1-{}_{1} \right) \right]\left( \frac{1}{D_{u}}+\Psi_{r} \right) \right)=\left( \frac{1}{L}+\Psi_{r} \right)\left( \frac{1}{D1}+\Psi_{r} \right)\left( \frac{1}{Du}+\Psi_{r} \right)$

S2 **The impact of variation in infectiousness between undetected and confirmed infections**

In Supplementary Table S2.1, we show and compare the results of modelling under three different values for the relative infectiousness (*ξ*) of undetected infections compared to that of confirmed cases under the assumption that the 1,290 deaths within Hubei province added on 17^th^ April 2020 were distributed before 20 Feb 2020.

It is shown that when undetected infections becomes less infectious comparing to confirmed cases, the early transmission coefficients (*β*_a_) increases, but the early transmissibility (*R*_0,1_) remains nearly the same; furthermore, the early ascertainment rate (*θ*_1,a_) and the contribution of undetected infections to *R*_0,1_ at early stage also remain at the similar levels.

Supplementary Table S2.1 Impact of relative infectiousness of undetected infections to the confirmed cases

| Name | Definition | Prior | Posterior | | |
| --- | --- | --- | --- | --- | --- |
|  |  |  | *ξ*=1 | *ξ*=1/2 | *ξ*=1/3 |
| *τ*_β_ | Turning time point in transmission rate | U[60,143]* | 70.6(69.2,71.9) | 70.7(70.0,71.9) | 71.3(69.1,72.0) |
| *β*_a_ | Transmission rate before *τ*_β_ | U[0.020,1.00] | 0.523(0.311,0.691) | 0.916(0.592,1.192) | 0.921(0.686,1.602) |
| *β*_b_ | Transmission rate after *τ*_β_ | U[0.001,0.20] | 0.009(0.001,0.031) | 0.012(0.002,0.034) | 0.011(0.002,0.050) |
| *R*_0,1_ | Reproduction number before *τ*_β_ | – | 2.33(1.96,3.69) | 2.20(1.94,3.11) | 2.16(1.79,3.21) |
| *R*_0_con_ | *R*_0_ due to confirmed cases before *τ*_β_ | – | 0.042(0.017,0.111) | 0.084(0.024,0.222) | 0.057(0.021,0.208) |
| *R*_0_und_ | *R*_0_ due to undetected cases before *τ*_β_ | – | 2.27(1.92,3.65) | 2.11(1.81,3.04) | 2.08(1.68,3.16) |
| *R*_0,2_ | Reproduction number after *τ*_β_ | – | 0.036(0.006,0.100) | 0.027(0.004,0.073) | 0.026(0.004,0.089) |
| *I*_0_ | Initial number of infectious people on 1/12/2019 | U[1,500] | 8.7(2.7,49.1) | 8.5(3.3,31.8) | 25.3(7.8,81.6) |
|  | Initial total number of people carrying the virus on 1/12/2019 | – | 40.9(12.8,207.2) | 43.0(16.8,167.3) | 142.0(18.0,444.8) |
| *τ*_θ_ | Turning time point in case ascertainment rate | U[40,62]* | 49.3(47.1,51.6) | 48.5(46.0,50.9) | 48.6(47.0,50.6) |
| *θ*_1,a_ | Case ascertainment rate before *τ*_θ_ | U[1%,25%] | 3.48%(1.74%,8.33%) | 4.22%(1.55%,10.17%) | 2.91%(1.17%,7.59%) |
| *θ*_1,b_ | Case ascertainment rate after *τ*_θ_ | U[25%,100%] | 36.61%(26.07%,55.39%) | 45.98%(31.54%,71.49%) | 43.90%(24.34%,80.20%) |
| *τ*_F_ | Turning time point in cCFR | U[65,95]* | 77.0(75.3,78.0) | 76.3(75.0,77.8) | 76.5(75.0,77.9) |
| *θ*_2,a_ | Fatality rate among confirmed cases (cCFR_1_) before *τ*_F_ | U[0.5%,50%] | 9.61%(8.12%,11.36%) | 10.78%(9.20%,12.60%) | 10.42%(9.10%,12.59%) |
| *θ*_2,b_ | Fatality rate among confirmed cases (cCFR_2_) after *τ*_F_ | U[0.1%,50%] | 0.67%(0.45%,0.97%) | 0.70%(0.47%,0.99%) | 0.68%(0.45%,1.03%) |
| *D*_1_ | Infectious period from symptom onset until hospitalization (days) | U[2.0,10] | 2.28(2.01,3.12) | 2.10(2.0,2.45) | 2.12(2.0,2.71) |
| *D*_1_*+D*_u_ | Infectious period of undetected infections (days) | U[3.0,25.0] | 4.44(3.19,11.71) | 4.82(3.31,10.31) | 6.93(3.37,13.84) |
| *η*^HOS^ | Dispersion parameter for confirmed cases | U[1.01,1500] | 76.0(50.2,122.8) | 88.1(57.6,152.8) | 93.1(55.7,162.0) |
| *η*^Death^ | Dispersion parameter for deaths | U[1.01,5000] | 3.5(2.4,5.2) | 3.9(2.6,6.0) | 3.7(2.5,6.3) |
| *η*^Recovery^ | Dispersion parameter for recoveries | U[1.01,1500] | 144.8(102.8,205.6) | 122.0(85.5,182.0) | 131.2(86.9,190.2) |
| IFR_1_ | Infection fatality rate (*θ*_1,a_*θ*_2,a_) before *τ*_θ_ | – | 0.33%(0.17%,0.85%) | 0.46%(0.16%,1.18%) | 0.30%(0.11%,0.85%) |
| IFR_2_ | Infection fatality rate (*θ*_1,b_*θ*_2,a_) between *τ*_θ_ and *τ*_F_ |  | 3.51%(2.60%,5.12%) | 5.01%(3.41%,7.79%) | 4.40%(2.71%,8.60%) |
| IFR_3_ | Infection fatality rate (*θ*_1,b_*θ*_2,b_) after *τ*_F_ | – | 0.24%(0.15%,0.41%) | 0.32%(0.19%,0.54%) | 0.27%(0.19%,0.55%) |

*: The epidemic was assumed to start from 1Dec 2019 (Huang *et al.* 2020)

**S3 Epidemic outbreaks in Hubei province and Wuhan city, China**

Hubei is a province in China with a population *N*=58.5 million and its capital city Wuhan with a population 11.0 million was the start place of the COVID-19 pandemic. Up to 21^st^ April 2020, 68,128 confirmed cases and 4,512 deaths were reported from Hubei province, and 50,333 confirmed cases and 3,869 deaths were reported within Wuhan city. In this part of supplementary information, we treat the outbreak within Hubei province as an independent transmission process and therefore ignore any exportation and importation from 1^st^ December 2019 to 21^st^ April 2020. For comparison, the outbreak within Wuhan city is similarly modelled as an independent transmission process. The synthesis model was applied to the epidemic outbreaks in Hubei province and in Wuhan respectively. The results of the model parameter estimation are listed in Supplementary Table S3.1 and the model fitting to data are shown in Figures S3.1 and S3.2.

Supplementary Table S3.1 Estimates of model parameters for the outbreak within Hubei province and the outbreak within Wuhan city, China

The 1,290 deaths within Hubei province added on 17^th^ April 2020 were distributed before 20 Feb 2020 in proportional to the daily number of deaths reported before 17^th^ April 2020. The relative infectiousness of undetected infections to confirmed cases *ξ* = 1 (i.e., both undetected and confirmed infections are of the same infectiousness). For comparison, the results for the model applied to the whole country are also listed.

| Name | Definition | Prior | posterior | | |
| --- | --- | --- | --- | --- | --- |
|  |  |  | Wuhan city | Hubei province | Mainland China |
| *τ*_β_ | Turning time point in transmission rate | U[60,143]* | 72.6(71.3,73.9) | 72.0(70.6,73.1) | 70.6(69.2,71.9) |
| *β*_a_ | Daily transmission rate before *τ*_β_ | U[0.020,1.00] | 0.517(0.332,0.685) | 0.454(0.264,0.650) | 0.523(0.311,0.691) |
| *β*_b_ | Daily transmission rate after *τ*_β_ | U[0.001,0.20] | 0.034(0.007,0.073) | 0.021(0.002,0.058) | 0.009(0.001,0.031) |
| *R*_0,1_ | Reproduction number before *τ*_β_ | – | 2.31(1.92,3.42) | 2.25(1.80,3.84) | 2.33(1.96,3.69) |
| *R*_0_con_ | *R*_0_ due to confirmed cases before *τ*_β_ | – | 0.032(0.011,0.112) | 0.027(0.009,0.110) | 0.042(0.017,0.111) |
| *R*_0_und_ | *R*_0_ due to undetected cases before *τ*_β_ | – | 2.27(1.90,3.37) | 2.21(1.78,3.80) | 2.27(1.92,3.65) |
| *R*_0,2_ | Reproduction number after *τ*_β_ | – | 0.127(0.044,0.227) | 0.084(0.020,0.178) | 0.036(0.006,0.100) |
| *I*_0_ | Initial number of infectious people on 1/12/2019 | U[1,500] | 5.3(2.3,27.9) | 19.8(2.3,75.1) | 8.7(2.7,49.1) |
|  | Initial total number of people carrying the virus on 1/12/2019 | – | 25.6(10.9,125.0) | 94.3(11.6,361.5) | 40.9(12.8,207.2) |
| *τ*_θ_ | Turning time point in case ascertainment rate | U[40,62]* | 47.2(44.2,49.8) | 49.1(46.5,50.9) | 49.3(47.1,51.6) |
| *θ*_1,a_ | Case ascertainment rate before *τ*_θ_ | U[1%,25%] | 2.78%(1.11%,8.43%) | 2.71%(1.13%,9.46%) | 3.48%(1.74%,8.33%) |
| *θ*_1,b_ | Case ascertainment rate after *τ*_θ_ | U[25%,100%] | 32.44%(25.34%,53.49%) | 31.75%(25.22%,58.18%) | 36.61%(26.07%,55.39%) |
| *τ*_F_ | Turning time point in cCFR | U[65,95]* | 77.2(76.0,78.0) | 77.4(76.1, 78.2) | 77.0(75.3,78.0) |
| *θ*_2,a_ | Fatality rate among confirmed cases (cCFR_1_) before *τ*_F_ | U[0.5%,50%] | 15.12%(13.31%,17.62%) | 11.46%(10.37%,13.54%) | 9.61%(8.12%,11.36%) |
| *θ*_2,b_ | Fatality rate among confirmed cases (cCFR_2_) after *τ*_F_ | U[0.1%,50%] | 0.74%(0.47%,1.12%) | 0.69%(0.47%,1.00%) | 0.67%(0.45%,0.97%) |
| *D*_1_ | Average infectious period from symptom onset until hospitalization (days) | U[2.0,10] | 2.19(2.01,2.95) | 2.18(2.01,2.93) | 2.28(2.01,3.12) |
| *D*_1_*+D*_u_ | Average infectious period of undetected infections (days) | U[3.0,25.0] | 4.52(3.16,10.03) | 5.07(3.15,13.24) | 4.44(3.19,11.71) |
| *η*^HOS^ | Dispersion parameter for reported cases | U[1.01,1500] | 146.7(73.7,322.1) | 148.0(77.6,322.0) | 76.0(50.2,122.8) |
| *η*^Death^ | Dispersion parameter for deaths | U[1.01,5000] | 4.0(2.8,5.8) | 3.3(2.3,4.9) | 3.5(2.4,5.2) |
| *η*^Recovery^ | Dispersion parameter for recoveries | U[1.01,1500] | 165.8(123.6,230.0) | 193.4(138.5,266.3) | 144.8(102.8,205.6) |
| IFR_1_ | Infection fatality rate (*θ*_1,a_*θ*_2,a_) before *τ*_θ_ | – | 0.43%(0.16%,1.33%) | 0.31%(0.13%,1.18%) | 0.33%(0.17%,0.85%) |
| IFR_2_ | Infection fatality rate (*θ*_1,b_*θ*_2,a_) between *τ*_θ_ and *τ*_F_ |  | 4.98%(3.71%,8.05%) | 3.69%(2.81%,6.65%) | 3.51%(2.60%,5.12%) |
| IFR_3_ | Infection fatality rate (*θ*_1,b_*θ*_2,b_) after *τ*_F_ | – | 0.24%(0.14%,0.44%) | 0.23%(0.14%,0.44%) | 0.24%(0.15%,0.41%) |

*: The epidemic was assumed to start from 1Dec 2019 (Huang *et al.* 2020)


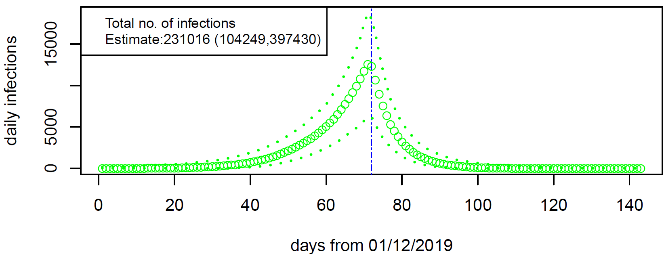

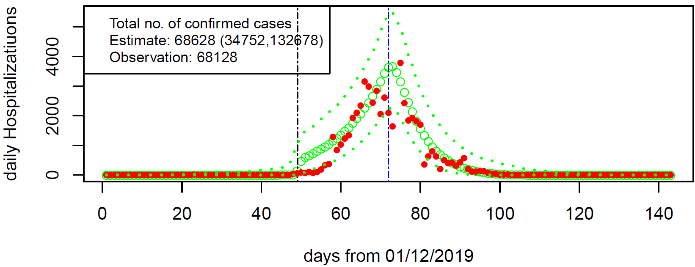

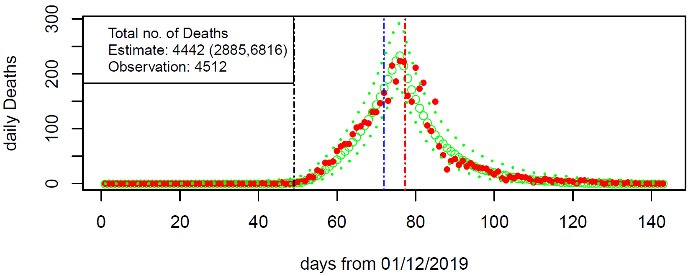

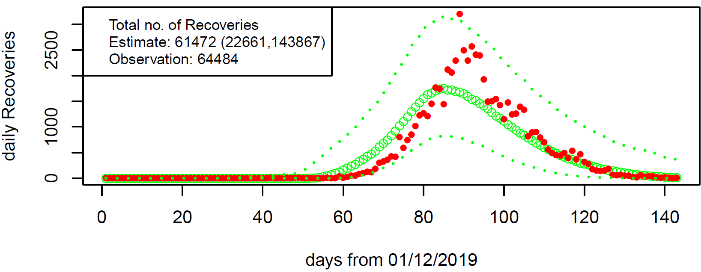


Supplementary Figure S3.1. Epidemic curves in Hubei province, China from 1^st^ December 2019 to 21^st^ April 2020. Model prediction of the number of infections (top left panel) and model fitting to the observations of confirmed cases (top right panel), deaths (bottom left panel) and recoveries (bottom right panel) are shown. The model predictions are obtained after redistributing the 1,290 deaths added on 17^th^ April 2020 to the period before 20^th^ February 2020 in accordance to the daily number of deaths reported before. The green dotted lines represent the model predictions (large green circles for median and thin green lines for lower and upper levels of 95% confidence interval). The red points are the observed data. The black, blue and red vertical lines denote the estimates of times when the case ascertainment rate, the transmission rate and confirmed case fatality rate respectively are estimated to have changed. Note that the daily number of confirmed cases (14,840) on 12^th^ February 2020 (day 74) is outside the top right panel.


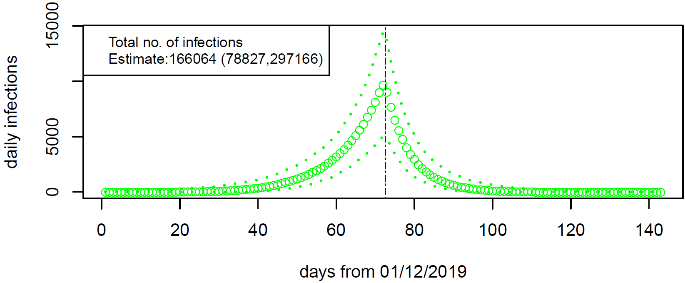
 **
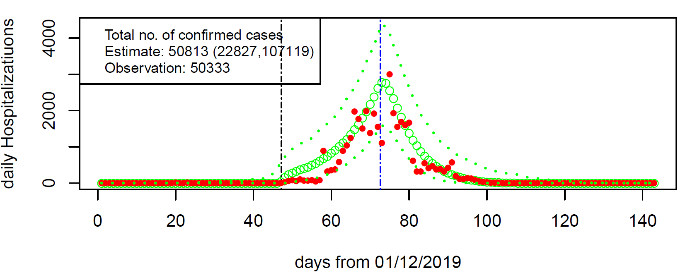

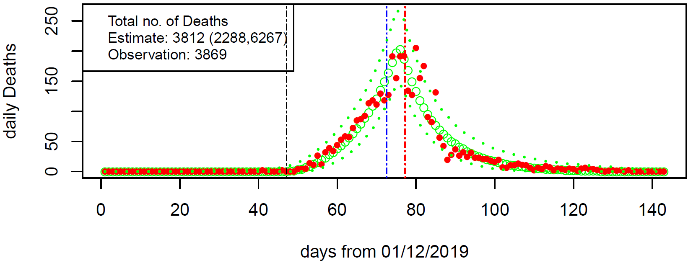

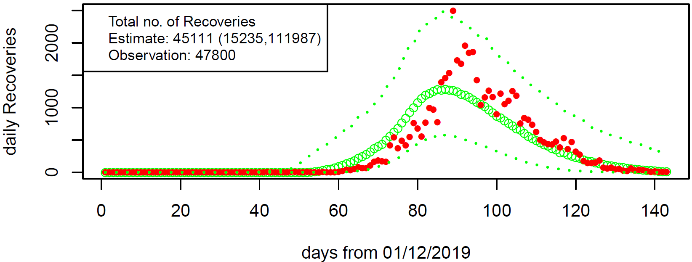
**

Supplementary Figure S3.2. Epidemic curves in Wuhan city, China from 1^st^ December 2019 to 21^st^ April 2020. Model prediction of the number of infections (top left panel) and model fitting to the observations of confirmed cases (top right panel), deaths (bottom left panel) and recoveries (bottom right panel) are shown. The model predictions are obtained after redistributing the 1290 deaths added on 17^th^ April 2020 to the period before 20^th^ February 2020 in accordance to the daily number of deaths reported before. The green dotted lines represent the model predictions (large green circles for median and thin green lines for lower and upper levels of 95% confidence interval). The red points are the observed data. The black, blue and red vertical lines denote the estimates of times when the case ascertainment rate, the transmission rate and confirmed case fatality rate respectively are estimated to have changed. Note that the daily number of confirmed cases (13,436) on 12^th^ February 2020 (day 74) is outside the top right panel.

**S4 Synthesis model under the assumption of pre-symptomatic transmission**

In the main text, we assume the latent period is equal to the incubation period, that is, the time of symptom onset occurs at the same time as people become infectious. In view of the evidence that the SARS-CoV-2 virus becomes infectious before the symptom onset (He *et al.* 2020; Savvides & Siegel 2020), in this part of supplementary information we divide the exposure compartment *E* into two equal parts: *E*_1_ and *E*_2_, and people in compartment *E*_2_ can pass the virus to other people with the same infectiousness as those in infection compartments *I*_1_ and *I*_u_. For simplicity, we consider the situation of *ξ* = 1 (i.e., both undetected and confirmed infections are of the same infectiousness)

The synthesis model is modified from equation (1) in the main text as

$$\frac{d}{dt}S\left( t \right)=-{\beta\left( t \right)S\left( t \right)\left( E_{2}\left( t \right)+I_{1}\left( t \right)+I_{u}\left( t \right) \right)}/N$$

$$\frac{d}{dt}E_{1}\left( t \right)={\beta\left( t \right)S\left( t \right)\left( E_{2}\left( t \right)+I_{1}\left( t \right)+I_{u}\left( t \right) \right)}/N-{{2E}_{1}\left( t \right)}/L$$

$$\frac{d}{dt}E_{2}\left( t \right)={{2E}_{1}\left( t \right)}/L-{{2E}_{2}\left( t \right)}/L$$

$\frac{d}{dt}I_{1}\left( t \right)={{2E}_{2}\left( t \right)}/L-{I_{1}\left( t \right)}/{D_{1}+Imported\left( t \right)}$

$\frac{d}{dt}I_{u}\left( t \right)=\frac{\left( 1-{}_{1} \right)I_{1}\left( t \right)}{D_{1}}-{I_{u}\left( t \right)}/{D_{u}}$

$$\frac{d}{dt}H\left( t \right)={{}_{1}I_{1}\left( t \right)}/{D_{1}}-{2_{2}H\left( t \right)}/{D_{\mathrm{Death}}-{2{(1-}_{2})H\left( t \right)}/{D_{\mathrm{Recovery}}}}$$

$$\frac{d}{dt}Recovered\_0(t)={2{(1-}_{2})H\left( t \right)}/{D_{\mathrm{Recovery}}}{-2Recovered\_0(t)}/{D_{\mathrm{Recovery}}}$$

$$\frac{d}{dt}Recovered\left( t \right)={2Recovered\_0(t)}/{D_{\mathrm{Recovery}}}$$

$$\frac{d}{dt}Dead\_0(t)={2_{2}H\left( t \right)}/{D_{\mathrm{Death}}-2Dead\_0}(t)/D_{\mathrm{Death}}$$

$$\frac{d}{dt}Dead\left( t \right)={2Dead\_0(t)}/{D_{\mathrm{Death}}}$$

At the early stage of our model system, the basic reproduction number *R*_0_ is given by

*R*_0,1_= (*D*_1_+(1-*θ*_1,a_)*D*_u_+*L*/2)*β*_a_

while at the late stage after the time point defined as max(τ_β_, τ_θ_),

*R*_0,2_= (*D*_1_+(1-*θ*_1,b_)*D*_u_+*L*/2)*β*_b_

Following the similar procedures in SI section 1, we can obtain the initial conditions given by the equations

$$E_{2}\left( 0 \right)=\frac{t/{D_{1}}+{}_{0}}{2t/L}I_{1}\left( 0 \right)$$

$$E_{1}\left( 0 \right)=\frac{2t/L+{}_{0}}{2t/L}E_{2}\left( 0 \right)$$

$I_{u}\left( 0 \right)=\frac{\left( 1-1 \right)t/{D_{1}}}{t/{D_{u}}+0}I_{1}\left( 0 \right)$

$$U\left( 0 \right)=\frac{t/{D_{u}}}{{}_{0}}I_{u}\left( 0 \right)$$

and *S*(0) = *N*-*E*_1_(0)- *E*_2_(0)-*I*_1_(0)-*I*_u_(0)-*U*(0). Here α_0_= exp(δt*Ψ*_r_)-1 for a small time step δ*t*.

The initial growth rate *Ψ*_r_ is linked to the transmission rate *β*_a_ through the following expression:

$\frac{{2\beta}_{a}}{L}\left( \left( \frac{1-{}_{1}}{D_{1}}+\frac{1}{D_{u}}+\Psi_{r} \right)\left( \frac{2}{L}+\Psi_{r} \right)+\left( \frac{1}{D1}+\Psi_{r} \right)\left( \frac{1}{Du}+\Psi_{r} \right) \right)=\left( \frac{2}{L}+\Psi_{r} \right)^{2}\left( \frac{1}{D1}+\Psi_{r} \right)\left( \frac{1}{Du}+\Psi_{r} \right)$

The estimates of model parameters when considering the pre-symptomatic transmission are listed in Supplementary Table S4.1. It shows that although the infectious periods of confirmed and undetected infections increase and the corresponding transmission coefficients (*β*_a_ and *β*_b_) decrease, the estimates of overall transmissibility (*R*_0_) and the confirmed case fatality rate remain roughly the same as that without pre-symptomatic transmission (Table 2 in the main text).

Supplementary Table S4.1 Estimates of model parameters for the COVID-19 outbreak within mainland China when considering the pre-symptomatic transmission

The 1,290 deaths within Hubei province added on 17^th^ April 2020 were distributed over the period before 20^th^ Feb 2020 in proportional to the daily number of deaths reported before 17^th^ April 2020. The relative infectiousness *ξ*=1 (i.e., both undetected and confirmed infections are of the same infectiousness) and the incubation period *L*=5.2 days. For comparison, the results for the model without pre-symptomatic transmission are also listed.

| Name | Definition | Prior | Posterior | |
| --- | --- | --- | --- | --- |
|  |  |  | With pre-symptomatic transmission | Without pre-symptomatic transmission |
| *τ*_β_ | Turning time point in transmission rate | U[60,143]* | 70.3(66.1,71.0) | 70.6(69.2,71.9) |
| *β*_a_ | Daily transmission rate before *τ*_β_ | U[0.020,1.00] | 0.249(0.199,0.296) | 0.523(0.311,0.691) |
| *β*_b_ | Daily transmission rate after *τ*_β_ | U[0.001,0.20] | 0.020(0.006,0.032) | 0.009(0.001,0.031) |
| *R*_0,1_ | Reproduction number before *τ*_β_ | – | 2.22(1.95,2.92) | 2.33(1.96,3.69) |
| *R*_0__con | R_0_ due to confirmed cases before *τ*_β_ | – | 0.033(0.014,0.132) | 0.042(0.017,0.111) |
| *R*_0__und | R_0_ due to undetected cases before *τ*_β_ | – | 2.19(1.92,2.85) | 2.27(1.92,3.65) |
| *R*_0,2_ | Reproduction number after *τ*_β_ | – | 0.15(0.06,0.23) | 0.036(0.006,0.100) |
| *I*_0_ | Initial number of infectious people on 1/12/2019 | U[1,500] | 9.6(1.2,134.7) | 8.7(2.7,49.1) |
|  | Initial total number of people carrying the virus on 1/12/2019 | – | 56.2(7.2,660.5) | 40.9(12.8,207.2) |
| *τ*_θ_ | Turning time point in case ascertainment rate | U[40,62]* | 49.3(47.2,51.3) | 49.3(47.1,51.6) |
| *θ*_1,a_ | Case ascertainment rate before *τ*_θ_ | U[1%,25%] | 2.71%(1.19%,8.00%) | 3.48%(1.74%,8.33%) |
| *θ*_1,b_ | Case ascertainment rate after *τ*_θ_ | U[25%,100%] | 35.80%(26.39%,51.56%) | 36.61%(26.07%,55.39%) |
| *τ*_F_ | Turning time point in cCFR | U[65,95]* | 77.1(76.0,78.0) | 77.0(75.3,78.0) |
| *θ*_2,a_ | Fatality rate among confirmed cases (cCFR_1_) before *τ*_F_ | U[0.5%,50%] | 9.53%(8.03%,11.18%) | 9.61%(8.12%,11.36%) |
| *θ*_2,b_ | Fatality rate among confirmed cases (cCFR_2_) after *τ*_F_ | U[0.1%,50%] | 0.70%(0.44%,1.10%) | 0.67%(0.45%,0.97%) |
| *D*_1_ | Average infectious period from symptom onset until hospitalization (days) | U[1.0,10] | 2.13(1.48,3.56) | 2.28(2.01,3.12) |
| *D*_1_*+L*/2 | Average infectious period of confirmed cases (days) | – | 4.73(4.08,6.12) | 2.28(2.01,3.12) |
| *D*_u_ | Average infectious period since symptom onset of undetected infections (days) | U[2.0,15.0] | 4.15(2.62,8.54) | 2.05(1.03,9.41) |
| *D*_1+_*D*_u_*+L*/2 | Total average infectious period of undetected infection (days) | – | 9.45(7.15,14.38) | 4.44(3.19,11.71) |
| *η*^HOS^ | Dispersion parameter for reported cases | U[1.01,1500] | 91.6(59.2,158.8) | 76.0(50.2,122.8) |
| *η*^Death^ | Dispersion parameter for deaths | U[1.01,5000] | 3.3(2.3,5.0) | 3.5(2.4,5.2) |
| *η*^Recovery^ | Dispersion parameter for recoveries | U[1.01,1500] | 154.8(106.2,228.8) | 144.8(102.8,205.6) |
| IFR_1_ | Infection fatality rate (*θ*_1,a_*θ*_2,a_) before *τ*_θ_ | – | 0.26%(0.11%,0.72%) | 0.33%(0.17%,0.85%) |
| IFR_2_ | Infection fatality rate (*θ*_1,b_*θ*_2,a_) between *τ*_θ_ and *τ*_F_ |  | 3.46%(2.34%,4.90%) | 3.51%(2.60%,5.12%) |
| IFR_3_ | Infection fatality rate (*θ*_1,b_*θ*_2,b_) after *τ*_F_ | – | 0.25%(0.15%,0.44%) | 0.24%(0.15%,0.41%) |

*: The epidemic was assumed to start from 1^st^ December 2019 (Huang *et al.* 2020)

**S5 Derivation of basic reproduction number *R*_0_ using next generation matrix**

In the following, we use the next generation matrix method described by Diekmann *et al.* (2010) to derive the expression of *R*_0_. The synthesis model described by equation (1) in the main text has three infected comparts, *E*, *I*_1_, and *I*_u_. At the infection-free steady state: *E*= *I*_1_= *I*_u_=0 and *S*= *N*. Linearizing the equations for the three compartments around the infection-free steady state (i.e., for the situation when *E*, *I*_1_, and *I*_u_ are small and ignoring any imported cases) gives rise to

$$\frac{d}{dt}E\left( t \right)={[I}_{1}\left( t \right)\left( {}_{1}+\left( 1-{}_{1} \right) \right)+I_{u}\left( t \right)]-{E\left( t \right)}/L$$

$\frac{d}{dt}I_{1}\left( t \right)={E\left( t \right)}/L-{I_{1}\left( t \right)}/{D_{1}}$

$\frac{d}{dt}I_{u}\left( t \right)=\left( 1-{}_{1} \right)I_{1}\left( t \right)/D_{1}-{I_{u}\left( t \right)}/{D_{u}}$

In accordance with Diekmann *et al.* (2010), we have the transmission matrix

$\mathbf{T}=\left( \begin{matrix} 0 & (_{1}+\left( 1-{}_{1} \right)) & \\ 0 & 0 & 0 \\ 0 & 0 & 0 \end{matrix} \right)$,

the transition matrix

$=\left( \begin{matrix} -\frac{1}{L} & 0 & 0 \\ \frac{1}{L} & -\frac{1}{D_{1}} & 0 \\ 0 & \frac{\left( 1-{}_{1} \right)}{D_{1}} & -\frac{1}{D_{u}} \end{matrix} \right)$,

and the next generation matrix as

$$\mathbf{K}=-\mathbf{T}{}^{\mathbf{-1}}$$

$=\left( \begin{matrix} 0 & (_{1}+\left( 1-{}_{1} \right)) & \\ 0 & 0 & 0 \\ 0 & 0 & 0 \end{matrix} \right)\left( \begin{matrix} L & 0 & 0 \\ D_{1} & D_{1} & 0 \\ \left( 1-{}_{1} \right)D_{u} & \left( 1-{}_{1} \right)D_{u} & D_{u} \end{matrix} \right)=\left( \begin{matrix} (_{1}+\left( 1-{}_{1} \right))D_{1}+\left( 1-{}_{1} \right)D_{u} & (_{1}+\left( 1-{}_{1} \right))D_{1}+\left( 1-{}_{1} \right)D_{u} & D_{u} \\ 0 & 0 & 0 \\ 0 & 0 & 0 \end{matrix} \right)$.

It is obvious that $(_{1}+\left( 1-{}_{1} \right))D_{1}+\left( 1-{}_{1} \right)D_{u}=[_{1}D_{1}+\left( 1-{}_{1} \right)(D_{1}+D_{u})]$ is its dominant eigenvalue and *R*_0_ of the synthesis model.

**Supplementary References:**

Diekmann, O., Heesterbeek, J.A.P., and Roberts, M.G. The construction of next generation matrices for compartmental epidemic models. *J. R. Interface* **7**, 873–885 (2010).

He, X. *et al*. Temporal dynamics in viral shedding and transmissibility of COVID-19 *Nat Med* <https://doi.org/10.1038/s41591-020-0869-5> (2020)

Huang, C. *et al*. Clinical features of patients infected with 2019 novel coronavirus in Wuhan, China. *Lancet*. **395**, 497–506. https://doi.org/10.1016/S0140-6736(20)30183-5 (2020)

Savvides, C. & Siegel, R. Asymptomatic and presymptomatic transmission of SARS-CoV-2: A systematic review. medRxiv preprint doi: <https://doi.org/10.1101/2020.06.11.20129072> (2020)

Wearing, H. J., Rohani, P., & Keeling M. J. Appropriate models for the management of

infectious diseases. PLoS Med 2(7):e174. (2005)
